# Supplementary material for: Quantification of a shelter cat population: Trends in intake, length of stay and outcome data of cats in seven Dutch shelters between 2006 and 2021
Source: PLoS One. 2023 May 19;18(5):e0285938. doi: 10.1371/journal.pone.0285938 (PMC10198509; doi:10.1371/journal.pone.0285938)

S1 File. Residual plots of the studied metrics to study the validity of the model about normality (left) and homoscedasticity (constant variability) (right).

Residual plots - Analysis of the average difference in total annual intake / 1000 residents (Fig 1a).

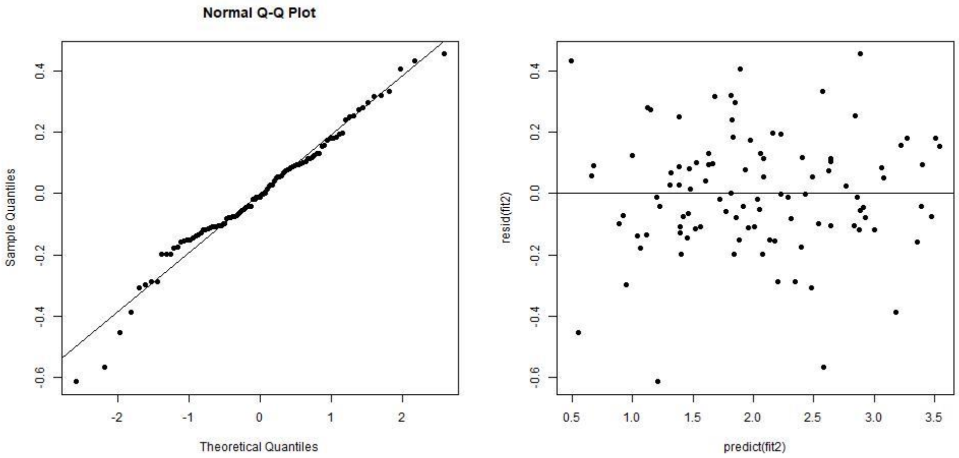

Residual plots - Analysis of the average difference in annual intake of stray cats / 1000 residents (Fig 3a).

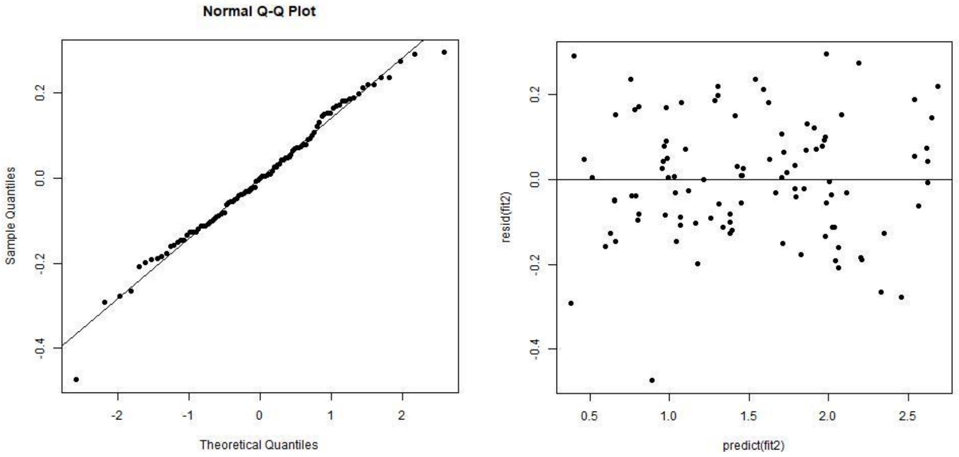

Residual plots - Analysis of the average difference in annual intake of owner surrendered cats (OSC) / 1000 residents (Fig 4a).

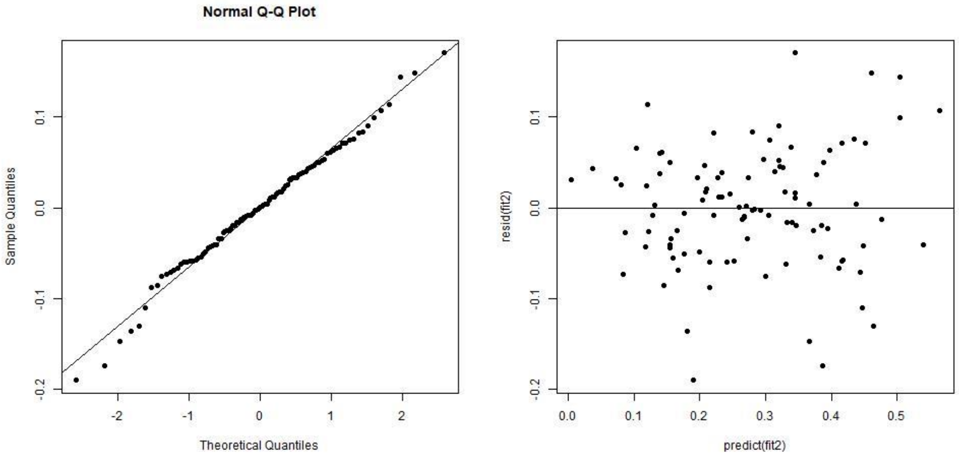

Residual plots - Analysis of the average difference in rehoming rate (RR) (Fig 6a).

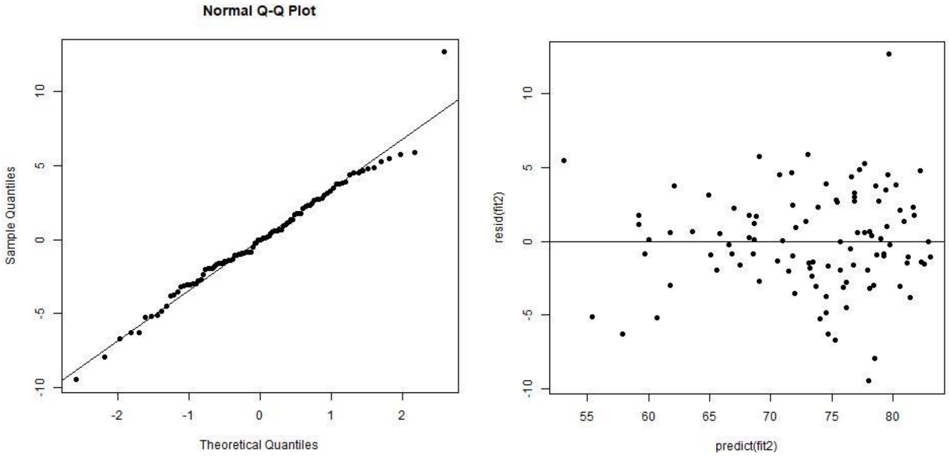

Residual plots - Analysis of the average difference in return to owner (RTO) (Fig 7a).

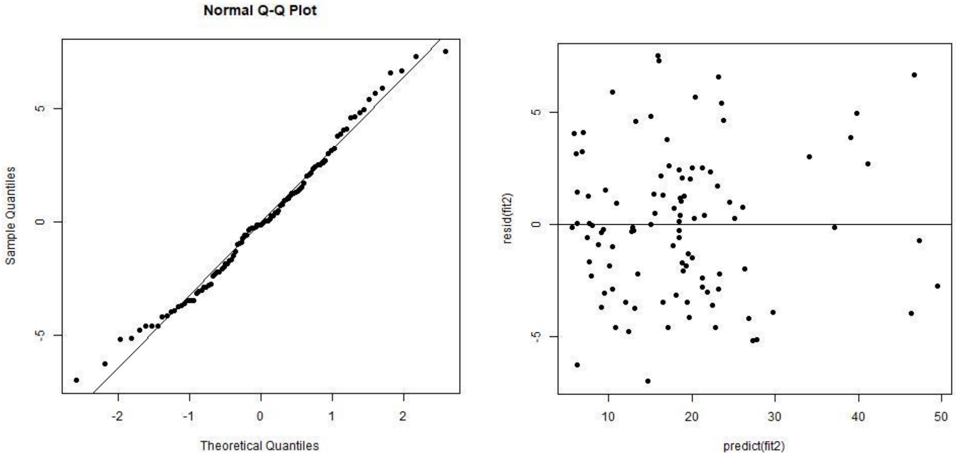

Residual plots - Analysis of the ratio of the mean difference in annual euthanasia cases / 1000 residents (Fig 8a).

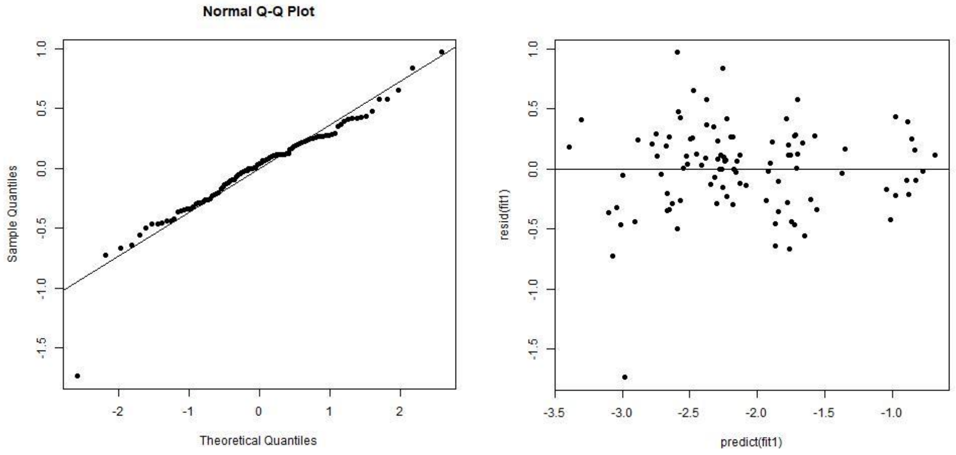

Residual plots - Analysis of the ratio of the mean length of stay (LOS) in total (Fig 9a).

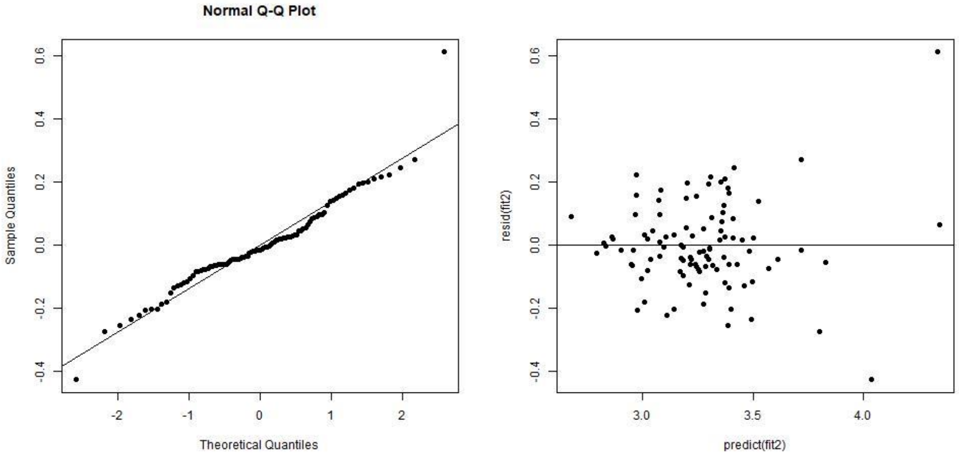

Residual plot - Analysis of the average difference in the mean length of stay (LOS) of stray cats (SC) (Fig 10a).

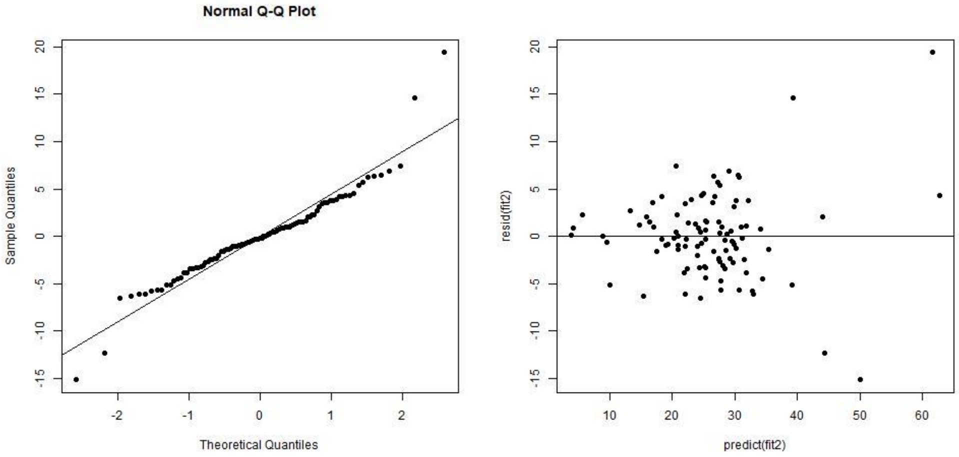

Residual plots - Analysis of the average difference in risk-based live release rate (RLRR) (Fig 11a).

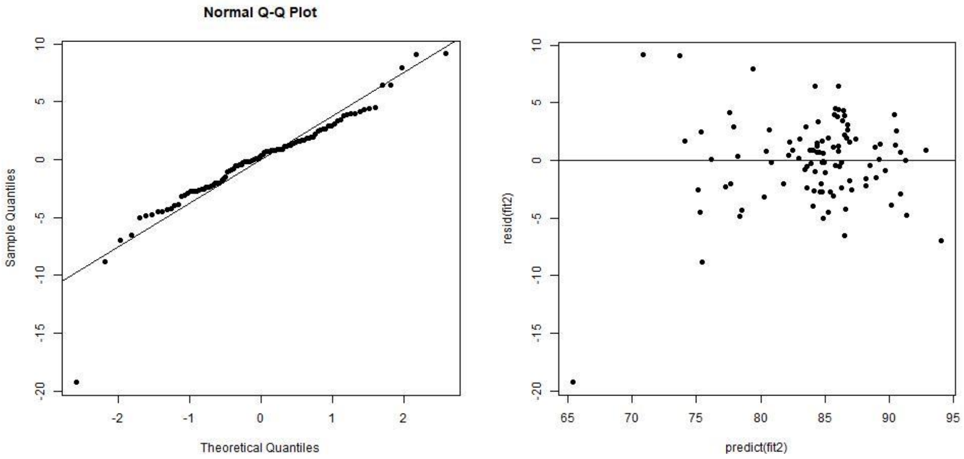

Residual plots - Analysis of the average difference in death rate (DR) (S3b Fig).

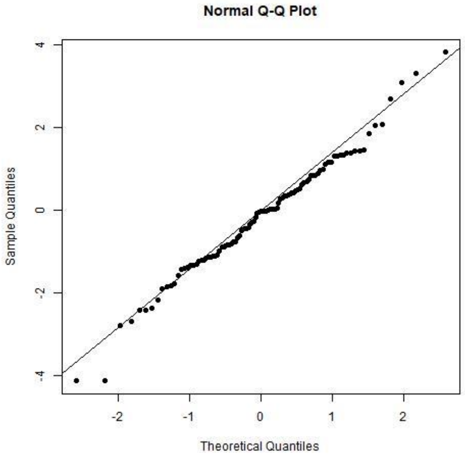

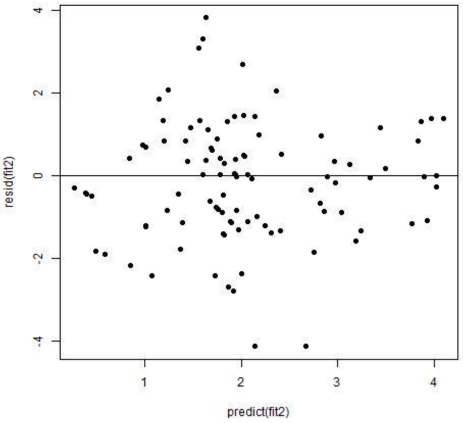

Supplement: S1 File — Residual plots of the studied metrics to study the validity of the model about normality and homoscedasticity (constant variability). (PDF) [file pone.0285938.s001.pdf]
