# Supplementary figures and images for: Quantification of a shelter cat population: Trends in intake, length of stay and outcome data of cats in seven Dutch shelters between 2006 and 2021
Source: PLoS One. 2023 May 19;18(5):e0285938. doi: 10.1371/journal.pone.0285938 (PMC10198509; doi:10.1371/journal.pone.0285938)

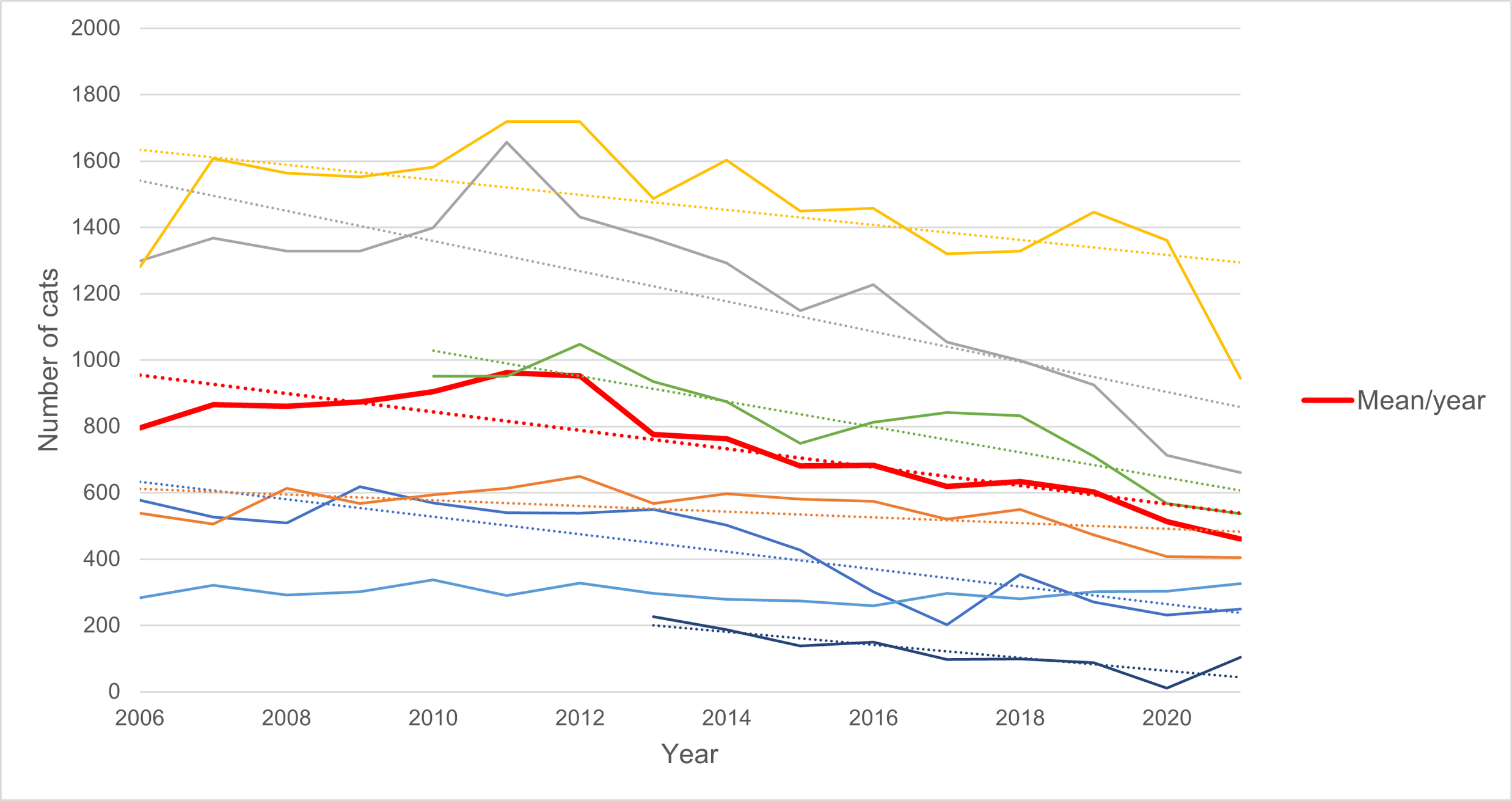

Supplement: S1 Fig — Annual total intake of cats per shelter. Annual data for seven shelters are used of which five shelters present data during the whole period of 2006–2021, six shelters during 2010–2021 and all seven shelters during 2013–2021. Regardless of shelter size, the total intake of cats decreased between 2006 and 2021 for all shelters in this study, shown also by the decreasing ’Mean / year’. (TIF) [file pone.0285938.s002.tif]

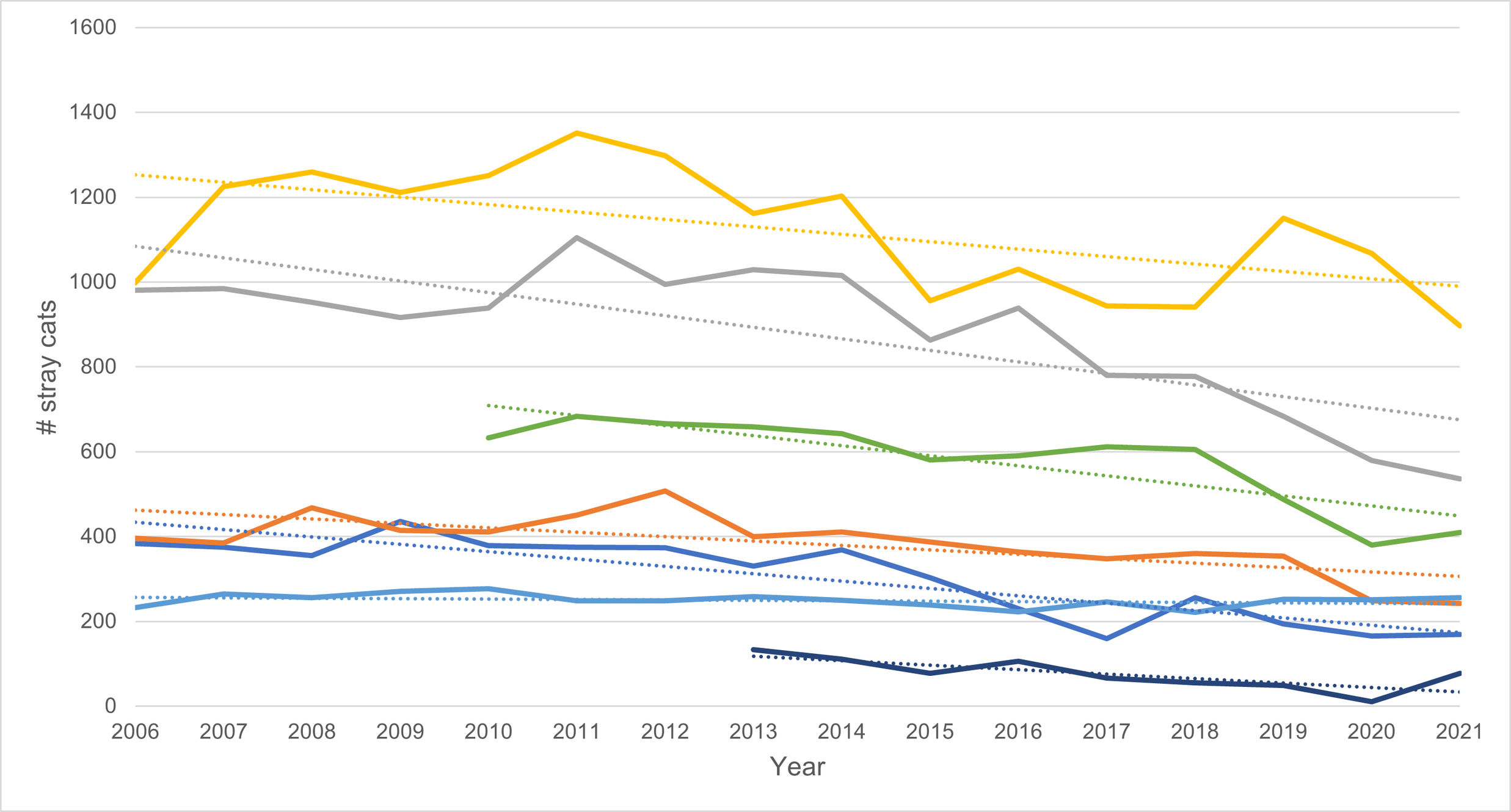

Supplement: S2 Fig — Annual intake of stray cats per shelter. Annual data for seven shelters are used of which five shelters present data during the whole period of 2006–2021, shelter F during 2010–2021 and shelter G during 2013–2021. Regardless of shelter size, the total intake of stray cats decreased between 2006 and 2021 for all shelters in this study. (TIF) [file pone.0285938.s003.tif]

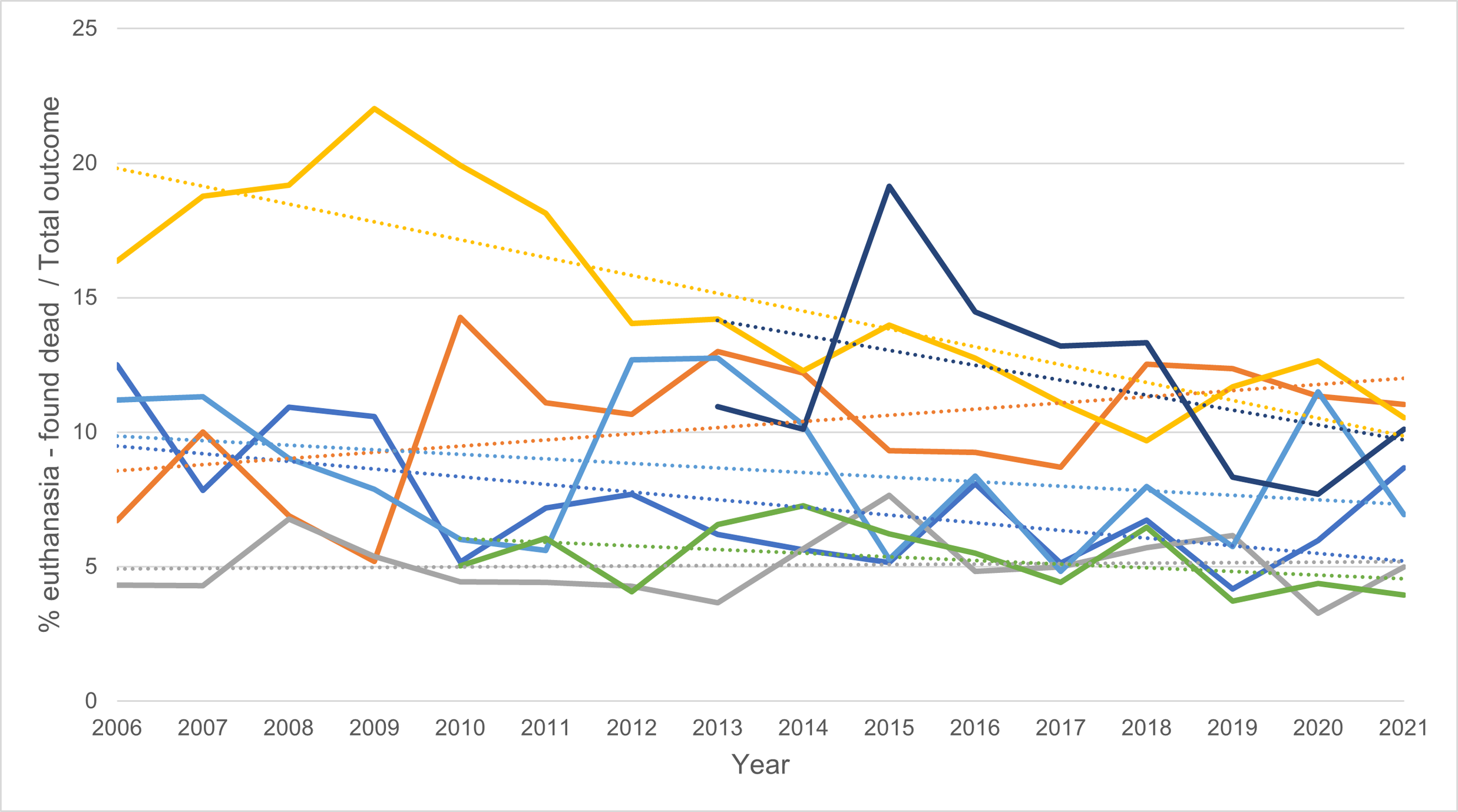

Supplement: S3 Fig — The annual feline cases of euthanasia and cats found dead as a proportion of the total outcome per shelter is pictured. One shelter had missing information between 2006 and 2009 and another shelter between 2006 and 2012. All seven shelters were included in the data for the years 2013 through 2021. (TIF) [file pone.0285938.s004.tif]

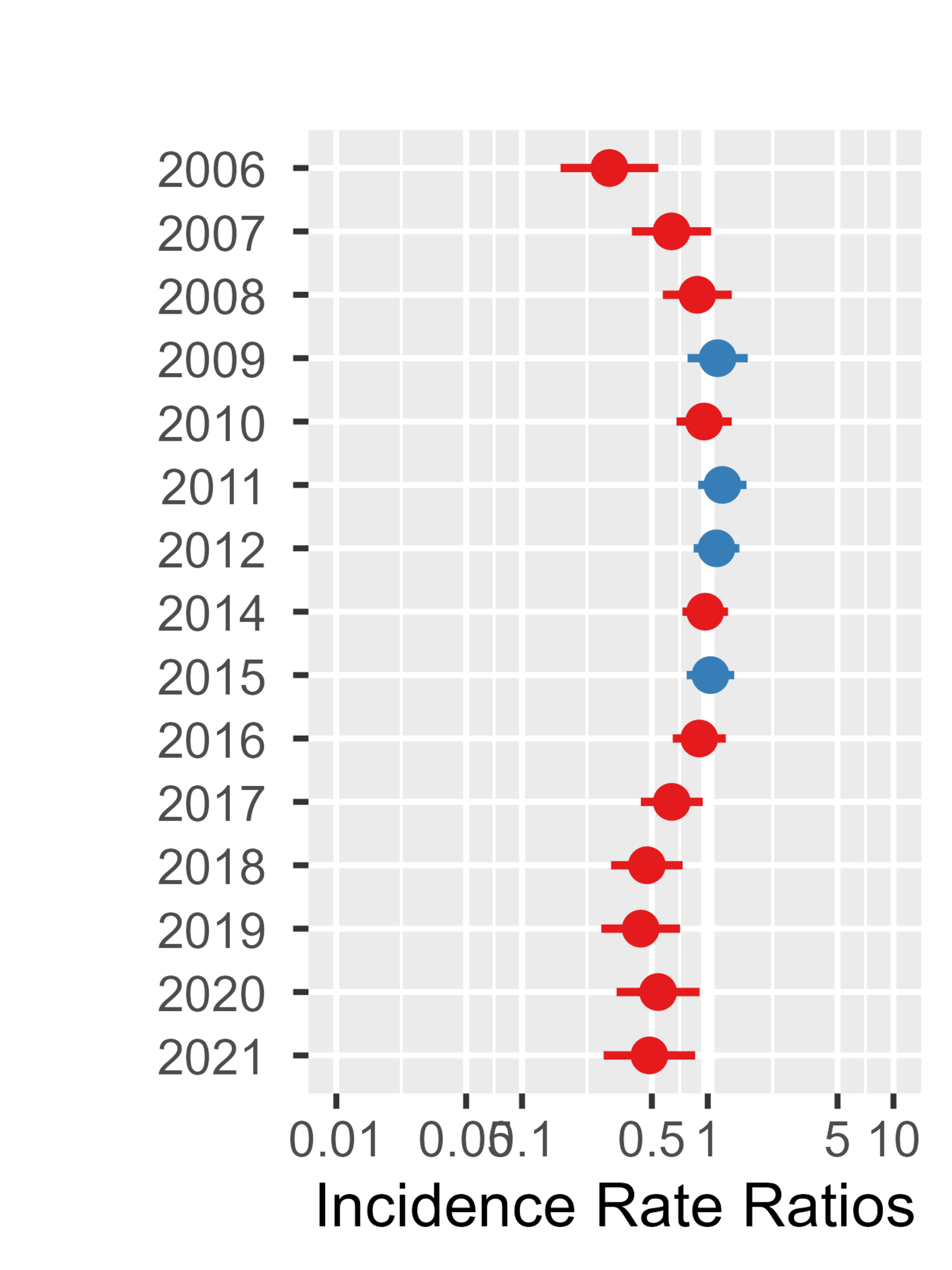

Supplement: S4 Fig — Estimates for the average differences in DR in seven shelters compared with 2013. The horizontal lines represent the estimate (the coloured dot) with 95% confidence intervals. An estimate of 0 (zero) means no difference from the annual DR in 2013. (TIF) [file pone.0285938.s005.tif]

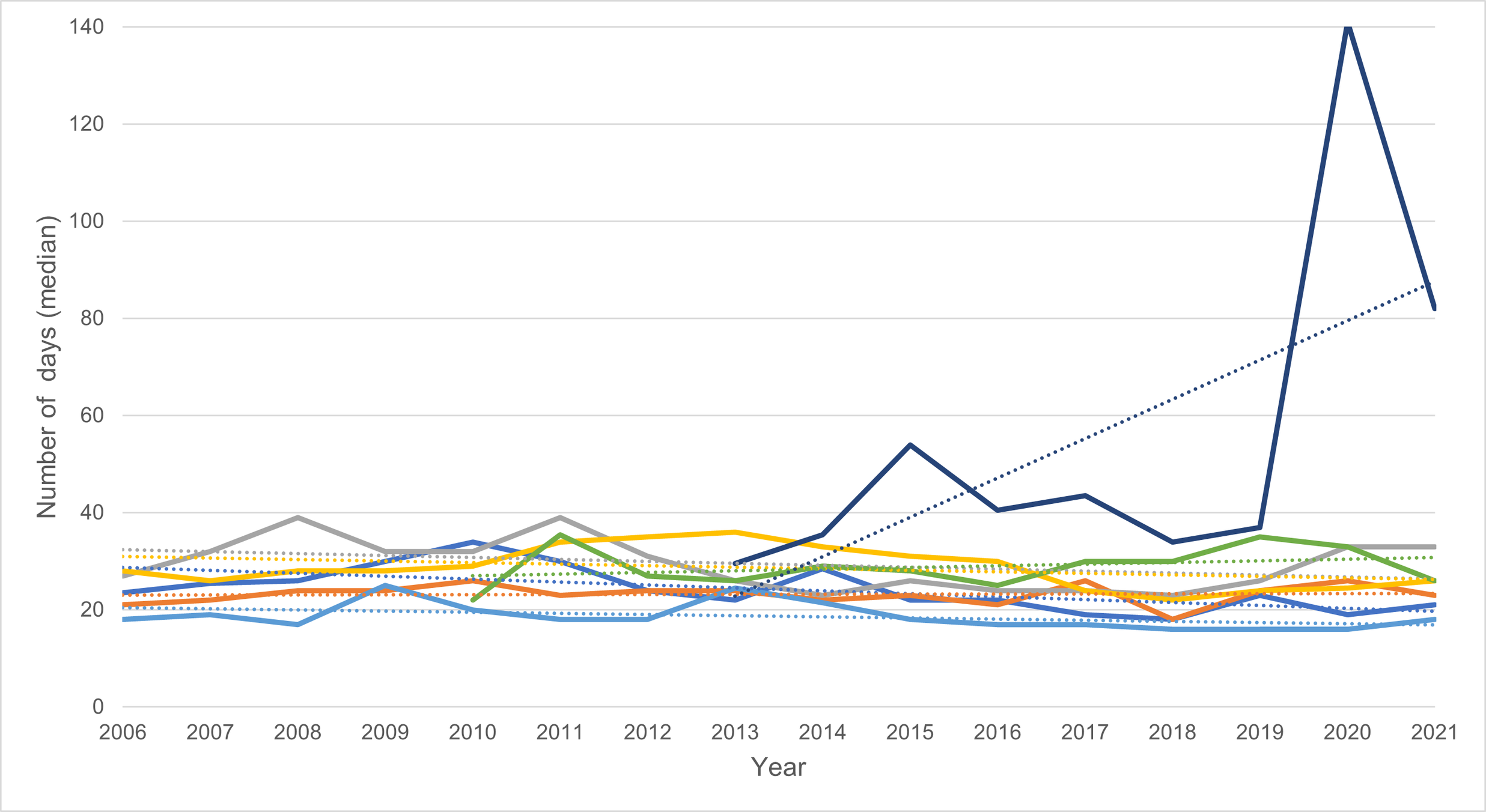

Supplement: S5 Fig — The median number of annual care days of all incoming cats per shelter is pictured. One shelter had missing information between 2006 and 2009 and another shelter between 2006 and 2012. All seven shelters were included in the data for the years 2013 through 2021. Data supplied by the smallest shelter in this study regarding the LOS (see darkest blue line) from 2020 and 2021 deviated considerably from the metrics of the other shelters. Given its limited size, alterations in the regular shelter management (for example during the SARS-CoV2 pandemic in 2020–2022) could have had a larger impact on its metrics compared with the larger shelters. (TIF) [file pone.0285938.s006.tif]

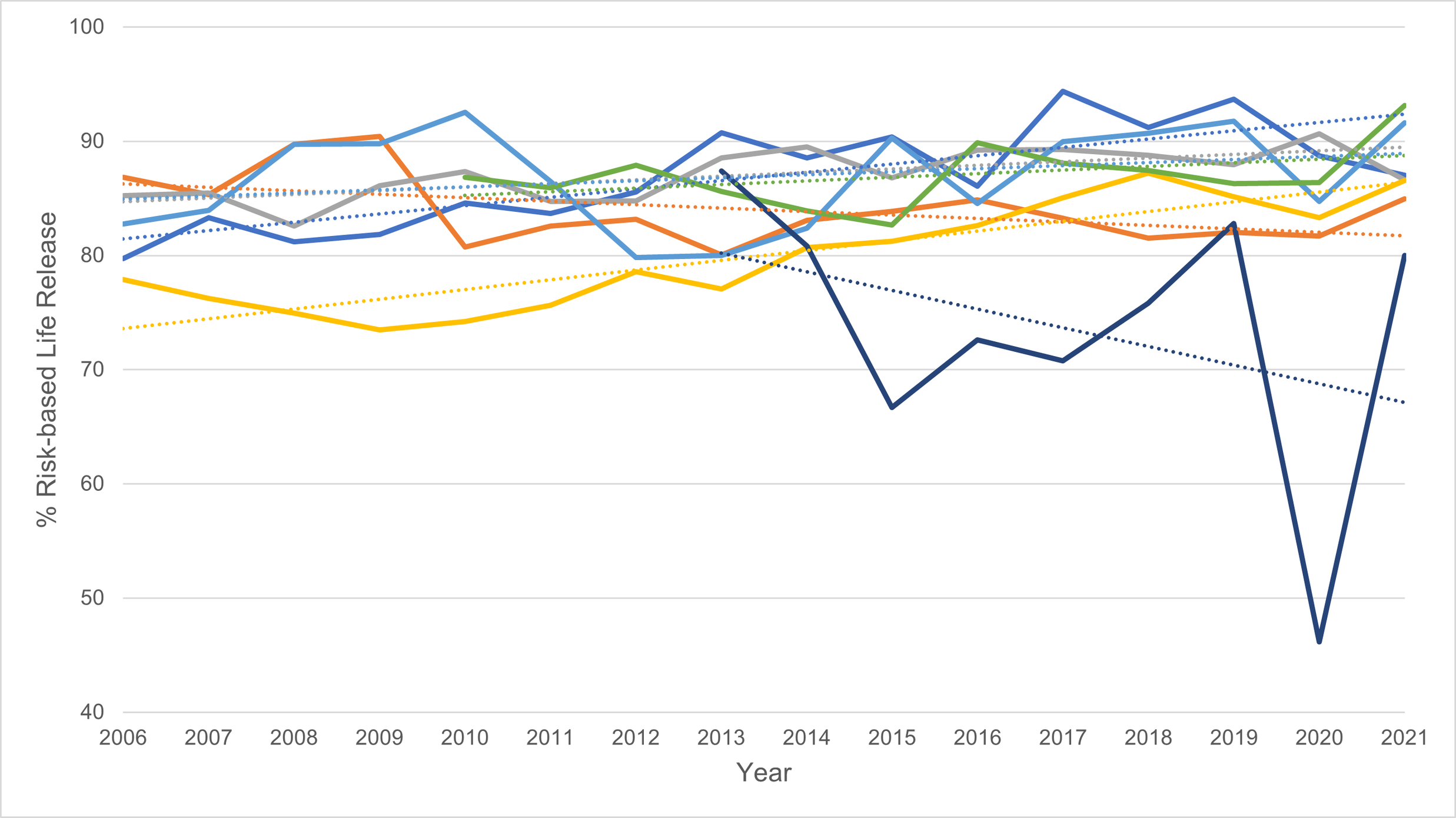

Supplement: S6 Fig — The annual RLRR of all cats is shown per shelter. One shelter had missing information between 2006 and 2009 and another shelter between 2006 and 2012. All seven shelters were included in the data for the years 2013 through 2021. Data supplied by the smallest shelter in this study regarding the RLRR (see darkest blue line) from 2020 and 2021 deviated considerably from the metrics of the other shelters. Given its limited size, alterations in the regular shelter management (for example during the SARS-CoV2 pandemic in 2020–2022) could have had a larger impact on its metrics compared with the larger shelters. (TIF) [file pone.0285938.s007.tif]
