# Supplementary material for: Quantification of a shelter cat population: Trends in intake, length of stay and outcome data of cats in seven Dutch shelters between 2006 and 2021
Source: PLoS One. 2023 May 19;18(5):e0285938. doi: 10.1371/journal.pone.0285938 (PMC10198509; doi:10.1371/journal.pone.0285938)
Supplement: S1 Table — (DOCX) [file pone.0285938.s008.docx]

**S1 Table. Characteristics of participating seven shelters.**

|  |  | **Annual intake of animal species per shelter** | | | **Characteristics human population in shelter care area** | |
| --- | --- | --- | --- | --- | --- | --- |
| **Shelter** | **Intake and Outcome data during years** | **Cats** | **Dogs** | **Other species** | **Income**  **per resident** | **Urbanisation rate** |
|  |  |  |  |  |  |  |
| **1** | 2006 - 2021 | 1464 | 340 | 0 | 25,400 | 4 |
| **2** | 2006 - 2021 | 1200 | 223 | 0 | 27,100 | 1 |
| **3** | 2006 - 2021 | 547 | 114 | 185 ^1^ | 28,300 | 3 |
| **4** | 2006 - 2021 | 448 | 59 | 0 | 28,800 | 2 |
| **5** | 2006 - 2021 | 298 | 49 | 0 | 24,000 | 4 |
| **6** | 2010 - 2021 | 817 | 97 | 0 | 29,300 | 2 |
| **7** | 2013 - 2021 | 122 | 10 | 9 ^2^ | 28,300 | 2 |

Information about the time range of shelter data for this study, their averaged annual feline intake (averaged over the total 16 years), the intake of dogs and other animal species (data for 2019), average annual income per resident in the shelter care area (data for 2019 [19]) and the urbanisation rate of the care area per shelter (1 = extremely urbanised area, 2 = strongly urbanised area, 3 = moderately urbanised area, 4 = hardly urbanised area, 5 = no urbanised area (classification according to the CBS – Classification of urbanisation [19]).

^1^ = rabbits, rodents, and birds. ^2^ = rabbits.
