# Supplementary material for: Quantification of a shelter cat population: Trends in intake, length of stay and outcome data of cats in seven Dutch shelters between 2006 and 2021
Source: PLoS One. 2023 May 19;18(5):e0285938. doi: 10.1371/journal.pone.0285938 (PMC10198509; doi:10.1371/journal.pone.0285938)
Supplement: S2 Table — (DOCX) [file pone.0285938.s009.docx]

**S2 Table. The estimated coefficients and their 95% confidence intervals for the intake of shelter cats.**

| **CATS INTAKE (per 1000 residents)** | | | | | | | | | |
| --- | --- | --- | --- | --- | --- | --- | --- | --- | --- |
|  | **CATS IN Total/1000 res.** | | | **Stray CATS IN Total/1000 res.** | | | **Surrender CATS IN Total/1000 res.** | | |
|  | **Confidence IntervaI** | | | **Confidence IntervaI** | | | **Confidence IntervaI** | | |
|  | **Estimate** | **2.5%** | **97.5%** | **Estimate** | **2.5%** | **97.5%** | **Estimate** | **2.5%** | **97.5%** |
| **Intercept** | 2.27 | 1.74 | 2.78 | 1.65 | 1.21 | 2.09 | 0.35 | 0.25 | 0.45 |
| **Year 2006** | -0.08 | -0.44 | 0.30 | -0.09 | -0.31 | 0.13 | 0.06 | -0.06 | 0.19 |
| **2007** | 0.13 | -0.20 | 0.47 | 0.07 | -0.14 | 0.28 | 0.05 | -0.07 | 0.16 |
| **2008** | -0.06 | -0.39 | 0.24 | -0.01 | -0.22 | 0.19 | -0.01 | -0.12 | 0.10 |
| **2009** | -0.06 | -0.39 | 0.24 | 0.07 | -0.14 | 0.27 | 0.06 | -0.04 | 0.16 |
| **2010** | 0.16 | -0.12 | 0.42 | 0.06 | -0.13 | 0.25 | 0.08 | 0.00 | 0.17 |
| **2011** | 0.18 | -0.07 | 0.46 | 0.12 | -0.06 | 0.31 | 0.07 | -0.01 | 0.15 |
| **2012** | 0.20 | -0.06 | 0.46 | 0.08 | -0.10 | 0.26 | 0.04 | -0.03 | 0.12 |
| **2014** | -0.10 | -0.34 | 0.14 | -0.03 | -0.20 | 0.14 | -0.05 | -0.13 | 0.02 |
| **2015** | -0.31 | -0.56 | -0.07 | -0.24 | -0.41 | -0.07 | -0.04 | -0.12 | 0.04 |
| **2016** | -0.34 | -0.59 | -0.07 | -0.22 | -0.40 | -0.05 | -0.07 | -0.15 | 0.01 |
| **2017** | -0.53 | -0.82 | -0.26 | -0.37 | -0.55 | -0.19 | -0.11 | -0.20 | -0.02 |
| **2018** | -0.50 | -0.80 | -0.20 | -0.38 | -0.56 | -0.19 | -0.12 | -0.21 | -0.02 |
| **2019** | -0.60 | -0.87 | -0.30 | -0.39 | -0.58 | -0.21 | -0.16 | -0.26 | -0.05 |
| **2020** | -0.88 | -1.23 | -0.55 | -0.60 | -0.79 | -0.40 | -0.18 | -0.30 | -0.06 |
| **2021** | -0.89 | -1.24 | -0.53 | -0.57 | -0.77 | -0.36 | -0.22 | -0.35 | -0.09 |

The estimated coefficients and their 95% confidence intervals of the linear mixed effect regression analysis of the variables: CATS IN Total/1000 residents, Stray CATS In Total/1000 residents and the Surrender Cats IN/1000 residents, for all seven shelters combined, with 'year' as explanatory factor. The annual human population in the shelter service area was used [19]. Year 2013 was taken as the reference year since all metrics were available from this year onwards for all shelters (one shelter had missing information between 2006 and 2009 and another shelter between 2006 and 2012). The estimates of these models should be interpreted as the difference between the mean number for a specific year compared with the mean number in year 2013.

Res. = residents.
