# Supplementary material for: Quantification of a shelter cat population: Trends in intake, length of stay and outcome data of cats in seven Dutch shelters between 2006 and 2021
Source: PLoS One. 2023 May 19;18(5):e0285938. doi: 10.1371/journal.pone.0285938 (PMC10198509; doi:10.1371/journal.pone.0285938)
Supplement: S3 Table — (DOCX) [file pone.0285938.s010.docx]

**S3 Table. The estimated coefficients and their 95% confidence intervals of the outcome of shelter cats.**

| **CATS OUTCOME** | | | | | | | | | |
| --- | --- | --- | --- | --- | --- | --- | --- | --- | --- |
|  | **Rehoming rate (% Total Outcome)** | | | **Return to Owner (% stray cats)** | | | **Log (Euthanasia/1000 res.)** | | |
|  | **Confidence IntervaI** | | | **Confidence IntervaI** | | | **Confidence IntervaI** | | |
|  | **Estimate** | **2.5%** | **97.5%** | **Estimate** | **2.5%** | **97.5%** | **Estimate** | **2.5%** | **97.5%** |
| **Intercept** | 72.69 | 67.59 | 77.78 | 18.35 | 12.06 | 24.63 | 0.14 | 0.08 | 0.24 |
| **Year 2006** | 2.48 | -2.12 | 7.07 | 0.09 | -6.17 | 6.35 | 1.13 | 0.70 | 1.81 |
| **2007** | 1.92 | -2.55 | 6.39 | -0.23 | -5.98 | 5.53 | 1.23 | 0.77 | 1.98 |
| **2008** | 3.33 | -1.03 | 7.70 | -3.06 | -8.35 | 2.22 | 0.92 | 0.58 | 1.49 |
| **2009** | 4.74 | 0.46 | 9.02 | -1.50 | -6.36 | 3.37 | 1.01 | 0.63 | 1.62 |
| **2010** | 5.36 | 1.37 | 9.36 | -2.08 | -6.38 | 2.21 | 1.04 | 0.67 | 1.64 |
| **2011** | 3.75 | -0.19 | 7.69 | -3.13 | -7.15 | 0.89 | 1.06 | 0.68 | 1.67 |
| **2012** | 1.22 | -2.69 | 5.13 | -2.77 | -6.61 | 1.06 | 1.07 | 0.68 | 1.68 |
| **2014** | -1.07 | -4.82 | 2.67 | 1.48 | -2.16 | 5.12 | 1.02 | 0.66 | 1.56 |
| **2015** | -1.27 | -5.05 | 2.50 | 1.83 | -1.99 | 5.65 | 0.93 | 0.60 | 1.42 |
| **2016** | -0.12 | -3.95 | 3.70 | 1.07 | -3.02 | 5.17 | 0.86 | 0.56 | 1.32 |
| **2017** | 2.57 | -1.32 | 6.47 | 0.78 | -3.67 | 5.24 | 0.62 | 0.40 | 0.95 |
| **2018** | -3.20 | -7.17 | 0.78 | 4.74 | -0.13 | 9.62 | 0.89 | 0.58 | 1.36 |
| **2019** | -0.10 | -4.19 | 3.98 | 3.88 | -1.47 | 9.22 | 0.63 | 0.41 | 0.98 |
| **2020** | 1.89 | -2.31 | 6.09 | 1.30 | -4.56 | 7.15 | 0.45 | 0.29 | 0.69 |
| **2021** | -3.61 | -7.94 | 0.72 | 2.87 | -3.52 | 9.26 | 0.49 | 0.32 | 0.76 |

The estimated coefficients and their 95% confidence intervals of the linear mixed effect regression analysis of the variables: ‘Rehoming rate' (as % of the Total Outcome), ‘Return to Owner' (as % of Stray Cats) and the ‘Euthanasia/1000 residents' for all seven shelters combined with 'year' as explanatory factor. For metrics ‘Euthanasia/1000 residents’ the metric was log transformed to meet the model assumptions. The annual human population in the shelter service area was used. Year 2013 was taken as the reference year since all metrics were available from this year onwards for all shelters (one shelter had missing information between 2006 and 2009 and another shelter between 2006 and 2012). The resulting estimates of the log transformed ‘Euthanasia/1000 residents' should be interpreted as a ratio: i.e., an estimate of 0.9 means that the mean number in the specific year is 0.9 times as large (e.g., 10% lower) compared to the mean number in the reference year 2013. The estimates of the non-transformed models should be interpreted as the difference between the mean number for a specific year compared to the mean number in year 2013.

Res. = residents.
