# Supplementary material for: Quantification of a shelter cat population: Trends in intake, length of stay and outcome data of cats in seven Dutch shelters between 2006 and 2021
Source: PLoS One. 2023 May 19;18(5):e0285938. doi: 10.1371/journal.pone.0285938 (PMC10198509; doi:10.1371/journal.pone.0285938)
Supplement: S4 Table — (DOCX) [file pone.0285938.s011.docx]

**S4 Table. The estimated coefficients and their 95% confidence intervals of the LOS and RLRR of shelter cats.**

| **CATS LOS and RLRR** | | | | | | | | | |
| --- | --- | --- | --- | --- | --- | --- | --- | --- | --- |
|  | **Log (LOS CATS Total Median)** | | | **LOS Strays (Median)** | | | **Risk based Life Release Rate** | | |
|  | **Confidence IntervaI** | | | **Confidence IntervaI** | | | **Confidence IntervaI** | | |
|  | **Estimate** | **2.5%** | **97.5%** | **Estimate** | **2.5%** | **97.5%** | **Estimate** | **2.5%** | **97.5%** |
| **Intercept** | 26.08 | 22.06 | 30.84 | 26.00 | 21.34 | 30.66 | 83.67 | 80.43 | 86.91 |
| **Year 2006** | 0.89 | 0.75 | 1.05 | -2.10 | -6.90 | 2.70 | -0.77 | -4.91 | 3.37 |
| **2007** | 0.94 | 0.80 | 1.11 | -1.27 | -5.79 | 3.26 | -0.42 | -4.40 | 3.55 |
| **2008** | 0.99 | 0.85 | 1.17 | 1.16 | -3.11 | 5.44 | 0.33 | -3.50 | 4.16 |
| **2009** | 1.07 | 0.91 | 1.25 | 1.20 | -2.87 | 5.26 | 1.02 | -2.69 | 4.72 |
| **2010** | 1.02 | 0.88 | 1.18 | 1.00 | -2.71 | 4.71 | 0.71 | -2.71 | 4.12 |
| **2011** | 1.11 | 0.96 | 1.28 | 3.17 | -0.41 | 6.74 | -0.50 | -3.85 | 2.84 |
| **2012** | 0.99 | 0.86 | 1.15 | 1.00 | -2.49 | 4.49 | -0.37 | -3.67 | 2.93 |
| **2014** | 0.99 | 0.86 | 1.14 | -0.67 | -4.16 | 2.83 | 1.01 | -2.29 | 4.31 |
| **2015** | 0.93 | 0.81 | 1.08 | -3.25 | -6.82 | 0.32 | 2.20 | -1.14 | 5.55 |
| **2016** | 0.88 | 0.76 | 1.01 | -4.50 | -8.21 | -0.79 | 2.52 | -0.89 | 5.94 |
| **2017** | 0.88 | 0.76 | 1.02 | -5.17 | -9.05 | -1.28 | 4.67 | 1.16 | 8.19 |
| **2018** | 0.79 | 0.68 | 0.92 | -6.67 | -10.77 | -2.57 | 4.14 | 0.51 | 7.77 |
| **2019** | 0.92 | 0.79 | 1.08 | -4.75 | -9.10 | -0.40 | 4.15 | 0.38 | 7.92 |
| **2020** | 0.94 | 0.80 | 1.10 | -3.33 | -7.96 | 1.29 | 2.24 | -1.69 | 6.18 |
| **2021** | 0.92 | 0.78 | 1.09 | -4.00 | -8.93 | 0.93 | 4.65 | 0.53 | 8.76 |

The estimated coefficients and their 95% confidence intervals of the linear mixed effect regression analysis of the variables: ‘Length of Stay (LOS) Cats Total', the ‘LOS of Stray Cats' and the ‘Risk based life release rate (RLRR)’, for all seven shelters combined with 'year' as explanatory factor. For metric ‘Length of Stay (LOS) Cats Total’ the metric was log transformed to meet the model assumptions. Year 2013 was taken as the reference year since all metrics were available from this year onwards for all shelters (one shelter had missing information between 2006 and 2009 and another shelter between 2006 and 2012). The resulting estimates of the log transformed ‘LOS CATS Total' should be interpreted as a ratio: i.e., an estimate of 0.9 means that the mean number in the specific year is 0.9 times as large (e.g., 10% lower) compared with the mean number in the reference year 2013. The estimates of the non-transformed models should be interpreted as the difference between the mean number for a specific year compared with the mean number in year 2013.

Res. = residents.
