# Supplementary material for: Quantification of a shelter cat population: Trends in intake, length of stay and outcome data of cats in seven Dutch shelters between 2006 and 2021
Source: PLoS One. 2023 May 19;18(5):e0285938. doi: 10.1371/journal.pone.0285938 (PMC10198509; doi:10.1371/journal.pone.0285938)
Supplement: Attachment — Submitted filename: Response to reviewers - May 2023.docx [file pone.0285938.s013.docx]

**Response to Reviewers**

Concerning: PONE-D-22-32536R1 - Quantification of a shelter cat population: trends in intake, length of stay and outcome data of cats in seven Dutch shelters between 2006 and 2021.

The lines referred to are the line numbers in the revised manuscript without track-changes.

**Comments of the academic editor:**

1- Editorial comments: As requested by reviewer 2, please consider having the text proofread by a native speaker, as the language quality needs improvement. This will ensure that the article meets the publication standards.

Our answer: the text was proofread and corrected by a native speaker.

2- Editorial comments: please include the graphics of the residual analyses as supplementary material.

Our answer: we added the graphics of the residual analyses as supplementary material as a **S1 File: Residual plots for the studied metrics.** Residual plots of the studied metrics to study the validity of the model about normality and homoscedasticity (constant variability).

3- Editorial comments: provide a brief explanation of why the linear mixed model was chosen and its properties. This will be useful for readers who are less familiar with the analysis.

Our answer: we added to the chapter ‘Statistics’ line 167 - 181:

For each shelter, the metrics were standardized and calculated per year. The metrics were repeatedly calculated and therefore correlated within the shelter. Although the metrics were standardized, the level of the metrics i.e., “CATS IN per year” for a specific shelter can be affected by its shelter management, finances etc. Simple linear regression models are based on linearity between outcome variable and continuous independent variable, normal distribution, and homoscedasticity (constant variability) of the residuals of independent observations. When the independent variable is categorical then this is called ANOVA and belongs to the family of linear regression models. The assumption of independence is violated in our data collection and the dependency between measurements of the same metric within shelter should be incorporated in the statistical model. We therefore used a linear mixed effects model which is a combination of estimation of fixed effects and so-called random effects i.e., estimation of variability between the independent subjects (shelter) as metrics were measured yearly within the same shelter. For comparison a very simple example of a model taking dependency of measurements into account is the paired t-test with two conditions within the same subject (i.e., body weight before and after diet treatment of the same person).
